# Unusual Upper Cervical Spine Disorder and Non-traumatic Atlantoaxial Rotatory Fixation in an Adult: A Case Report

**DOI:** 10.7759/cureus.75051

**Published:** 2024-12-03

**Authors:** Fuminori Kamakura, Shota Shimizu, Keisuke Shigenobu, Gaku Yasuda, Yoshimasa Ishigaki, Satoshi Goto

**Affiliations:** 1 Department of Orthopedic Surgery, Fujimi-Kogen Hospital, Fujimi-Kogen Medical Center, Fujimi, JPN; 2 Department of Orthopedic Surgery, Shinshu University School of Medicine, Matsumoto, JPN; 3 Department of Orthopedic Surgery, Suwa Central Hospital, Chino, JPN

**Keywords:** adult, atlantoaxial rotatory fixation (aarf), conservative treatment, fielding classification, non-traumatic

## Abstract

This study reports on an extremely rare case of non-traumatic atlantoaxial rotatory fixation (AARF) in an adult. Although there are numerous reports on traumatic AARF in adults, those on non-traumatic AARFs are limited. We present the case of a 25-year-old woman who developed neck pain with a limited range of motion (ROM) that began upon waking without any particular inducement. Physical examination showed a characteristic torticollis neck posture called the cock robin position. The patient exhibited severely limited neck mobility with no neurological deficits. Computed tomography (CT) revealed that the atlas was rotated to the right in relation to the axis, resulting in a diagnosis of AARF Fielding type 1. Conservative treatment was adopted by immobilizing the cervical spine using a soft neck collar. Four weeks post-treatment, all symptoms improved with no adverse complications. We present an extremely rare case of non-traumatic AARF in an adult patient. As surgical treatment may be necessary if the diagnosis is delayed, appropriate diagnosis and treatment should be made in the early stages.

## Introduction

Atlantoaxial rotatory fixation (AARF) is a disorder of the upper cervical spine wherein the intervertebral joint between the atlas and axis becomes rotated and fixed [1]. It occurs more frequently in children with symptoms of acute neck pain and a limited range of motion (ROM) in the neck, exhibiting a characteristic torticollis posture [2]. Atlantoaxial rotatory fixation is generally caused by trauma to the head and neck or an upper respiratory infection [3]. Atlantoaxial rotatory fixation rarely occurs in adults, and almost all cases are a result of trauma. Although traumatic AARF is rare, numerous cases have been reported; however, non-traumatic AARF without any inducement has almost never been reported.

Moreover, non-traumatic AARF in adults is almost non-existent. Here, we present an extremely rare case of non-traumatic AARF along with a comprehensive review of relevant literature. Given our findings, we contend that non-traumatic AARF should be considered as a new differential diagnosis for torticollis in adults.

## Case presentation

**Figure 1:**
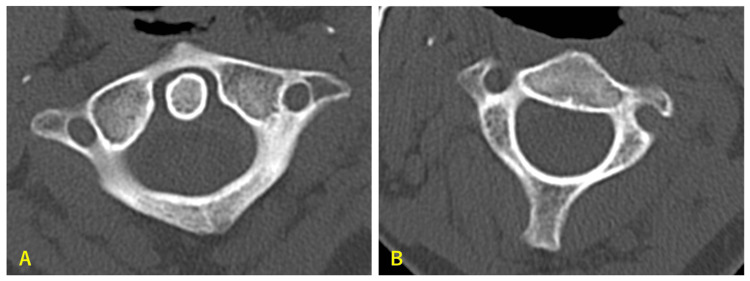
Axial scan using computed tomography (CT) The atlas is rotated approximately 20 degrees to the right (A) relative to the axis (B) and fixed. No expansion of the atlantodental interval (ADI) is seen.

**Figure 2:**
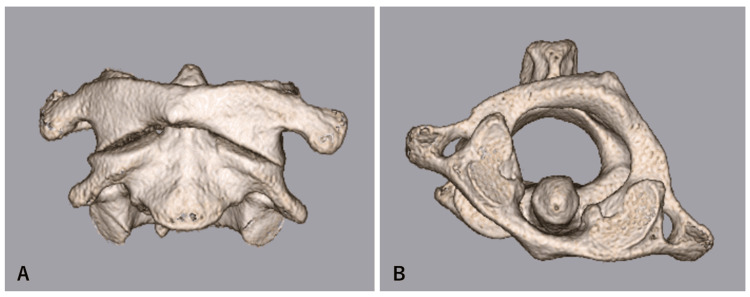
Three-dimensional computed tomography (3D-CT) of the atlas and axis The atlas and axis are viewed from the front (A) and from above (B). In addition to the plain CT (Figure 1), the atlas is rotated to the right relative to the axis and fixed. No expansion of the atlantodental interval (ADI) is observed.

A 25-year-old female office worker developed neck pain and limited ROM of the neck without any particular reason upon waking and visited our hospital four days later. She reported no history of recent infections or traumatic events. Physical examination revealed a characteristic torticollis neck posture, and the patient demonstrated severely limited neck mobility. The patient showed no neurological deficits. Computed tomography (CT) revealed an atlas rotated and fixed in the right direction in relation to the axis (Figures 1, 2).

**Figure 3:**
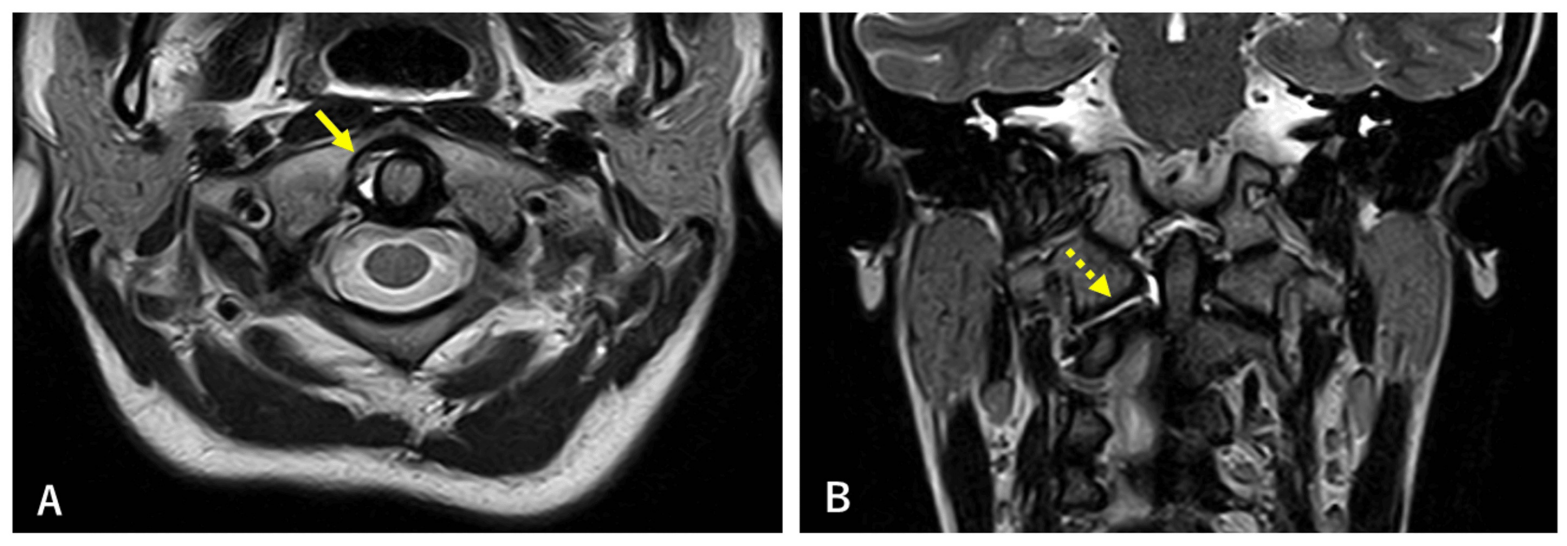
T2-weighted imaging of magnetic resonance imaging (MRI) There is a small amount of effusion on the right side of the odontoid (Figure 3a, arrow) and right facet joint (Figure 3b, broken arrow). No damage to the transverse ligament or alar ligament was observed.

Magnetic resonance imaging (MRI) showed effusion on the right side of the odontoid process and right intervertebral joint at C1/2, with no damage to the transverse ligament or alar ligament (Figure 3).

There were no imaging findings suggestive of local infection, bony deformation, or a neoplastic lesion. The patient was subsequently diagnosed with AARF (Fielding type 1). Conservative treatment was adopted by immobilizing the cervical spine using a soft neck collar on the day of diagnosis, and follow-up was performed in an outpatient setting. Non-steroidal anti-inflammatory drugs (NSAIDs) and opioids were prescribed to reduce pain. Four weeks post-treatment, the patient regained full neck mobility without any pain or adverse complications, and the collar was successfully removed.

## Discussion

Atlantoaxial rotatory fixation was first classified by Fielding and Hawkins in 1977 [1]. Patients with AARF exhibit a characteristic torticollis posture, also known as the "cock robin" position [2]. Atlantoaxial rotatory fixation is generally caused by trauma to the head and neck or an upper respiratory infection [3]. In children, AARF generally occurs after trauma to the head and neck or an upper respiratory infection [3]. However, almost all published AARF cases in adult patients were caused by trauma or some external force [3,4]. In children, a combination of factors, including a large head, underdeveloped neck musculature, a rotational angle greater than 45 degrees, the horizontal configuration of the C1-2 articular facets, and increased elasticity of the joint capsules, make it easier to cause AARF [5]. To the best of our knowledge, only Isogai et al. have reported a non-traumatic AARF case without any inducement in adults [6]. Their patient was initially treated with Glisson’s traction, which failed, after which closed reduction under general anesthesia was performed successfully [6]. Fortunately, the present case was treated only using immobilization with a soft neck collar, and the patient recovered without any adverse effects.

Grisel’s syndrome is another upper cervical disease similar to AARF that rarely occurs in adults [7]. However, it differs from AARF in terms of pathophysiology, occurring after head and neck surgery, upper respiratory tract infection, or the inflammation of adjacent soft tissues [7]. Thus, it is also important to consider Grisel’s syndrome in the differential diagnosis of upper cervical deformation.

Computed tomography, particularly three-dimensional CT (3D-CT), is useful for evaluating the relationship between the atlas and the axis of the spine [8]. The Fielding classification is widely used to evaluate the degree of displacement of the atlas into four patterns, known as the atlantodental interval (ADI) [1]. Magnetic resonance imaging (MRI) can also be used to assess transverse and alar ligaments [9].

Immobilization, Glisson’s traction, manual reduction, and operative fixation are the selected treatments depending on the situation [3]. Katsuyama et al. provide a treatment flowchart for adult AARF [3]. They investigated 32 adult AARF patients and found that patients who were Fielding type 1 diagnosed within one month could be treated conservatively, using immobilization, traction, and manual reduction; conversely, all patients diagnosed more than one month after injury or who were Fielding type 2, 3, or 4 failed to improve after conservative treatment and underwent surgery [3]. Therefore, an early diagnosis is essential for successful conservative treatment [3]. In the present case, the appropriate diagnosis of AARF at the acute stage and early treatment with immobilization may have led to a successful prognosis.

The strengths of this case report include the rarity of the case itself and the favorable prognosis achieved through early diagnosis and prompt initiation of treatment. However, the limitations include the short follow-up period of just four weeks, which may not capture long-term outcomes, and the inability to completely rule out the recurrence of AARF as a result of the limited follow-up duration.

## Conclusions

We presented an extremely rare case of non-traumatic AARF in an adult patient. Computed tomography and MRI were useful to evaluate local conditions in detail. As surgical treatment may be necessary if the diagnosis is delayed, an appropriate diagnosis must be made in the early stages, and treatment should be initiated as soon as possible.
